# Recruitment maneuver does not provide any mortality benefit over lung protective strategy ventilation in adult patients with acute respiratory distress syndrome: a meta-analysis and systematic review of the randomized controlled trials

**DOI:** 10.1186/s40560-018-0305-9

**Published:** 2018-06-26

**Authors:** Sulagna Bhattacharjee, Kapil D. Soni, Souvik Maitra

**Affiliations:** 1 Department of Anaesthesiology, Pain Medicine and Critical Care, All India Institute of Medical Sciences, Room No. 5011, 5th Floor Teaching block, Ansari Nagar New Delhi, 110029 India; 2 Department of Trauma Critical Care, Jai Prakash Narayan Apex Trauma Centre, All India Institute Medical Sciences, New Delhi, India

**Keywords:** ARDS, Recruitment maneuver, Open lung, PEEP titration

## Abstract

**Background:**

Clinical benefits of recruitment maneuver in ARDS patients are controversial. A number of previous studies showed possible benefits; a large recent study reported that recruitment maneuver and PEEP titration may even be harmful. This meta-analysis was designed to compare the clinical utility of recruitment maneuver with low tidal volume ventilation in adult patients with ARDS.

**Methods:**

Randomized controlled trials comparing recruitment maneuver and lung protective ventilation strategy with lung protective strategy ventilation protocol alone in adult patients with ARDS has been included in this meta-analysis. PubMed and Cochrane Central Register of Controlled Trials were searched from inception to 10 November 2017 to identify potentially eligible trials. Pooled risk ratio (RR) and standardized mean difference (SMD) were calculated for binary and continuous variables respectively.

**Results:**

Data of 2480 patients from 7 randomized controlled trials have been included in this meta-analysis and systemic review. Reported mortality at the longest available follow-up [RR (95% CI) 0.93 (0.80, 1.08); *p* = 0.33], ICU mortality [RR (95% CI) 0.91 (0.76, 1.10); *p* = 0.33] and in-hospital mortality [RR (95% CI) 0.95 (0.83, 1.08); *p* = 0.45] were similar between recruitment maneuver group and standard lung protective ventilation group. Duration of hospital stay [SMD (95% CI) 0.00 (− 0.09, 0.10); *p* = 0.92] and duration of ICU stays [SMD (95% CI) 0.05 (− 0.09, 0.19); *p* = 0.49] were also similar between recruitment maneuver group and standard lung protective ventilation group. Risk of barotrauma was also similar.

**Conclusion:**

Use of recruitment maneuver along with co-interventions such as PEEP titration does not provide any benefit in terms of mortality, length of ICU, and hospital stay in ARDS patients.

**Electronic supplementary material:**

The online version of this article (10.1186/s40560-018-0305-9) contains supplementary material, which is available to authorized users.

## Background

Acute respiratory distress syndrome (ARDS) is a potentially life-threatening hypoxic respiratory failure, characterized by arterial hypoxemia (PaO_2_/FiO_2_ < 200), pulmonary congestion, and decreased respiratory compliance [1] Single centric studies reported a wide range of incidence of ARDS in intensive care unit (ICU) patients [2]. A large international multicenter observation study [3] in 2016 reported that incidence of ARDS was more than 10% in all ICU patients, and it was over 23% in all patients requiring mechanical ventilation. Reported unadjusted ICU mortality and hospital mortality in that study were 35.3 and 40%, respectively.

Atelectasis from alveolar or interstitial edema and consolidation and intra-pulmonary shunt are important pathophysiologic basis hypoxemia in ARDS patients [4]. Increased pulmonary capillary permeability from a variety of pulmonary and extra-pulmonary insults causes pulmonary edema in these patients [5]. Atelectasis contributes to the ventilator-induced lung injury by reducing the amount of functional aerated lung unit and repeated recruitment and de-recruitment of the small alveoli increases sheer stress leading to atelectotrauma [6]. Recruitment maneuver includes elevations of applied airway pressure for short duration aiming to recruit the collapsed alveoli and increase the number of alveolar units participating in tidal ventilation [7]. Positive end-expiratory pressure (PEEP) helps to keep the recruited lung unit ‘open’ and thereby reduces atelectasis and improves oxygenation [8]. Recruitment maneuver is usually used along with other methods of open lung approach such as high PEEP. Recruitment maneuver provides short-term improvement in oxygenation and lung compliances; on the contrary, it may be associated with barotrauma from increased airway pressure and hemodynamic compromise [6].

We designed this systematic review and meta-analysis of randomized controlled trials to know the clinical benefits of recruitment maneuver alone or along with other therapeutic modalities of open lung approach such as high PEEP or PEEP titration in adult patients with ARDS.

## Methods

This meta-analysis follows the recommendations of Preferred Reporting Items for Systematic Review and Meta-Analysis Protocols (PRISMA- P) statement [9]. A protocol of this meta-analysis has not been registered.

### Eligibility criteria

Published prospective randomized controlled trials comparing recruitment maneuver and lung protective ventilation strategy with lung protective strategy ventilation protocol in adult patients with ARDS has been included in this meta-analysis. Trials where PEEP titration was used following recruitment maneuver were also considered for inclusion in this meta-analysis. Trials of those that did not report mortality data for at least a one-time point and where a lung protective ventilation strategy has not been used have been considered to be included in this meta-analysis.

### Information sources

PubMed and The Cochrane Library databases (CENTRAL) were searched for potentially eligible trials from inception to 10 November 2016. We have not imposed any language restriction or date restriction in search strategy. References of the previously published meta-analyses were also searched for eligible trials.

### Search strategy

The following keywords were used to search database: “ARDS, acute respiratory distress syndrome, acute lung injury, acute hypoxemic respiratory failure, recruitment maneuver, recruitment manoeuvre, lung recruitment, open lung.” Details of PubMed search strategy have been provided in Additional file 1.

### Study selection

Two authors (SM and KDS) independently searched title and abstract of the potentially eligible articles. Finally, full text of the possible articles was retrieved and assessed for eligibility. Any disputes between the two authors were solved by discussion and consultation with a third author (SB).

### Data collection process

Two authors (SM and SB) independently retrieved required data from the eligible RCTs, and all data were initially tabulated in a Microsoft Excel™ (Microsoft Corp., Redmond, WA) data sheet. Another author crosschecked these data before analysis (KDS).

### Data items

The following data were retrieved from the full text for all studies: first author, year of publication, country where work was done, sample size, characteristics of included patients, respiratory goals (oxyhemoglobin saturation, arterial oxygen, and PaO_2_/FiO_2_), details of recruitment maneuver (method application, any associated therapeutic modality, timing of recruitment maneuver and duration, details of rescue therapy, if any), details of mechanical ventilation, and clinical outcome (reported complications, organ dysfunction, length of hospital and ICU stay, and mortality at different time points).

### Risk of bias in individual studies

Two authors (SM and SB) independently assessed the methodological quality of the included studies. The following methodological questions were searched from the studies as per the Cochrane methodology: method of randomization, allocation concealment, blinding of the participants and personnel, blinding of outcome assessment, incomplete data reporting, selective reporting, and any other bias. For each area of bias, we will designate the trials as low risk of bias, unclear risk of bias, or high risk of bias. Risk of bias at individual study level will be graphically presented in the review.

### Summary measures and synthesis of results

Primary outcome of this meta-analysis is ‘mortality at longest available follow-up’ in the included patients. Secondary outcomes are ICU mortality rate, in-hospital mortality rate, incidence of barotrauma after randomization, incidence of hemodynamic compromise after randomization and length of hospital and ICU stay.

For continuous variables, mean and standard deviation (SD) values were extracted for both groups, a standardized mean difference (SMD) was computed at the study level, and a weighted mean difference was computed in order to pool the results across all studies. If the values were reported as median and an inter-quartile range or total range of values, the mean value was estimated using the median and the low and high end of the range for samples smaller than 25; for samples greater than 25, the median itself was used. The standard deviation (SD) was estimated from the median, and the low and high end of the range for samples smaller than 15, as range/4 for samples from 15 to 70, and as range/6 for samples more than 70. If only an inter-quartile range was available, SD was estimated as inter-quartile range/1.35 [10].

For binary outcomes, we calculated the following: [1] the risk ratio (RR) for each trial; [2] the pooled RR using the inverse variance method; [3] the number needed to treat (NNT) where a statistical significance was found, i.e., the number of patients who must be treated for one patient to benefit from the intervention. NNT was calculated from OR in Visual Rx online software (Visual Rx version 3.0, Dr. Chris Cates, http://www.nntonline.net/visualrx/). All statistical variables were calculated with 95% confidence interval (95% CI). The Q-test was used to analyze heterogeneity of trials. Considering possible clinical heterogeneity due to study design and patients’ population, we used a random effect model for all pooled analysis. Pooled analysis was done in RevMan software (Review Manager (RevMan) [Computer program]. Version 5.3. Copenhagen: The Nordic Cochrane Centre, The Cochrane Collaboration, 2014). Publication bias was assessed by visual inspection of funnel plot. A meta-regression was planned by *metareg* command in STATA version 13.0 (STATA SE 13.0, Stata Corp, College Station, TX, USA) in case of more than 10 trials is found for any outcome.

## Results

**Fig. 1:**
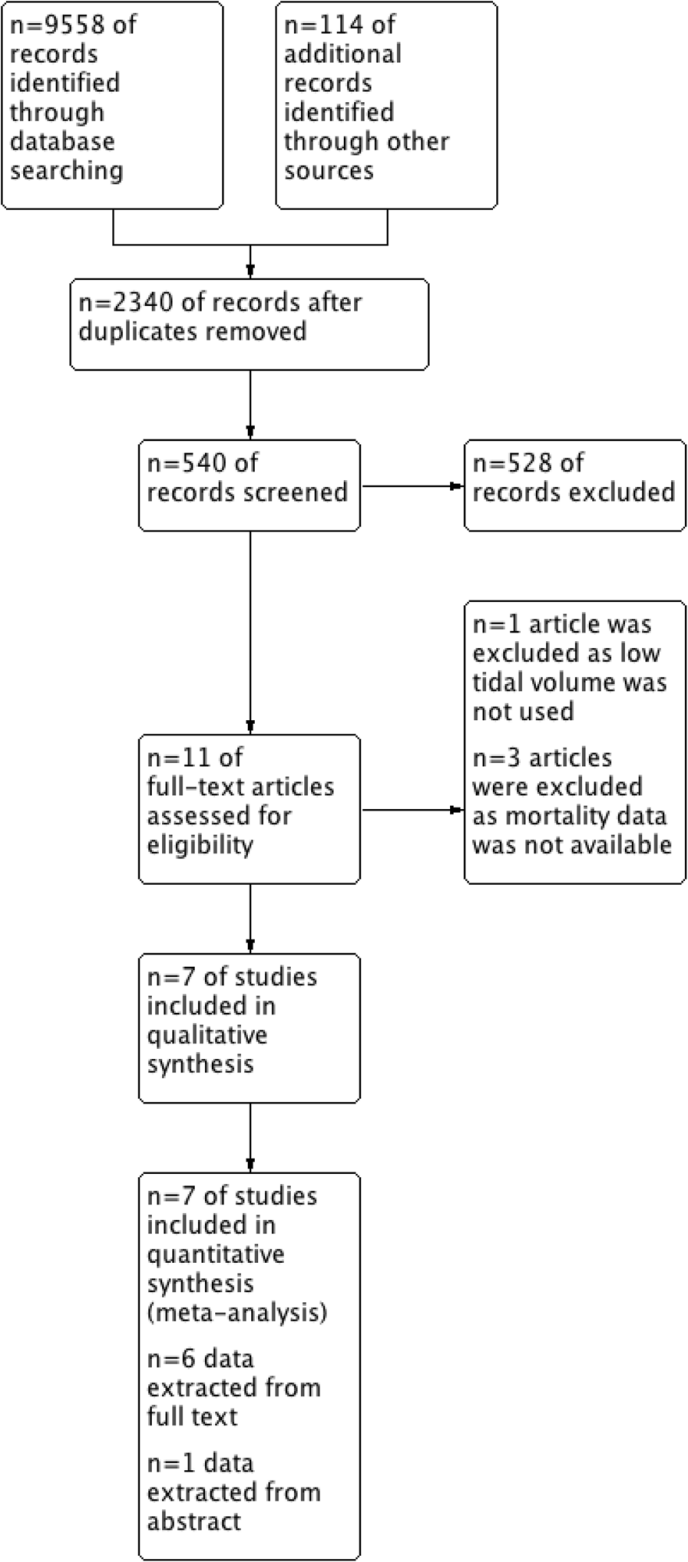
Flow diagram showing stages of database searching and study selection

**Fig. 2:**
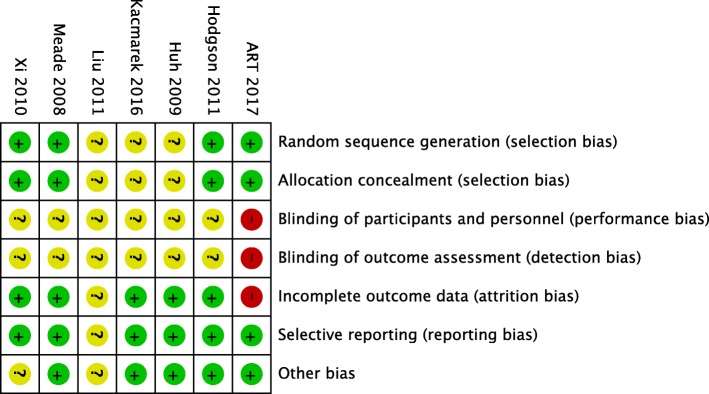
A summary of risk of biases showing review authors’ judgments about each risk of bias item for each included study

Initial searching of database revealed 9558 articles, and searching of the other sources revealed another 114 articles. After duplicate removal, 540 articles were assessed and 12 articles were screened from title and abstract to identify potentially eligible trials. Finally, data of 2480 patients from 7 randomized controlled trials from published full text [11–16] and abstract [17] have been included in this meta-analysis and systemic review. A flow diagram showing stages of database searching and study selection has been provided in Fig. 1. One RCT [18] was excluded as lung protective ventilation was not used in control group and three trials [19–21] were excluded, as they did not report mortality data. Risk of biases in the individual studies have been reported in Fig. 2. Characteristics of the individual studies have been reported in Table 1.

**Table 1:** Characteristics of the included studies

Author	Participants	Intervention	Control
ART investigators 2017	Patients receiving invasive mechanical ventilation with moderate to severe ARDS (AECC definition) of < 72 h duration	LRM with incremental PEEP levels, followed by decremental PEEP titration according to the best respiratory system Cs and by a second LRM	Low PEEP strategy
Hodgson 2011	Adult patients (age > 15years) with PaO_2_/FiO_2_ < 200	LRM to *P*_peak_ of 55 cm H_2_O and decremental PEEP titration to determine optimal PEEP	ARDSnet protocol
Kacmarek 2016	ARDS patients with PaO_2_/FiO_2_ < 200 at FiO_2_ ≥ 0.5 and PEEP ≥ 10	LRM to *P*_peak_ of ≤ 60 cm H_2_O for 2 min and decremental PEEP titration to determine optimal PEEP.	ARDSnet protocol
Liu 2011	Adult patients with ARDS (PaO_2_/FiO_2_ ≤ 250 mmHg) with FiO_2_ ≥ 0.5 and PEEP ≥ 10 cm H_2_O) at least 30 min	LRM with PEEP 35 cm H_2_O and *P*_peak_ up to 50 cm H_2_O maintained for 2 min, then PEEP was set higher at 2 cm H_2_O above closing pressure	Lung protective ventilation strategy
Xi 2010	Adult patients with ARDS (PaO_2_ ≤ 200 mmHg at FiO_2_ 1.0 and PEEP ≥ 10 cm H_2_O)	LRM with CPAP 40 cm H_2_O for 40 s	Lung protective ventilation strategy
Meade 2008	Adult patients with ARDS (PaO_2_/FiO_2_ < 250)	LRM by CPAP of 40 cm H_2_O for 40 s with FiO_2_ 1.0. PEEP was adjusted as per FiO_2_ requirement.	Low tidal volume ventilation with standard PEEP
Huh 2009	ARDS patients with PaO_2_/FiO_2_ < 200	LRM to *P*_peak_ of 55 cm H_2_O and 25% tidal volume reduction and decremental PEEP titration to determine optimal PEEP	ARDSnet protocol

*ARDS* acute respiratory distress syndrome, *AECC* American European Consensus Conference Criteria, *PaO*_*2*_ arterial oxygen tension, *FiO*_*2*_ fraction of oxygen in inspiration, *PEEP* positive end-expiratory pressure, *P*_*peak*_ peak airway pressure, *LRM* lung recruitment maneuver, *CPAP* continuous positive airway pressure

### Mortality

**Fig. 3:**
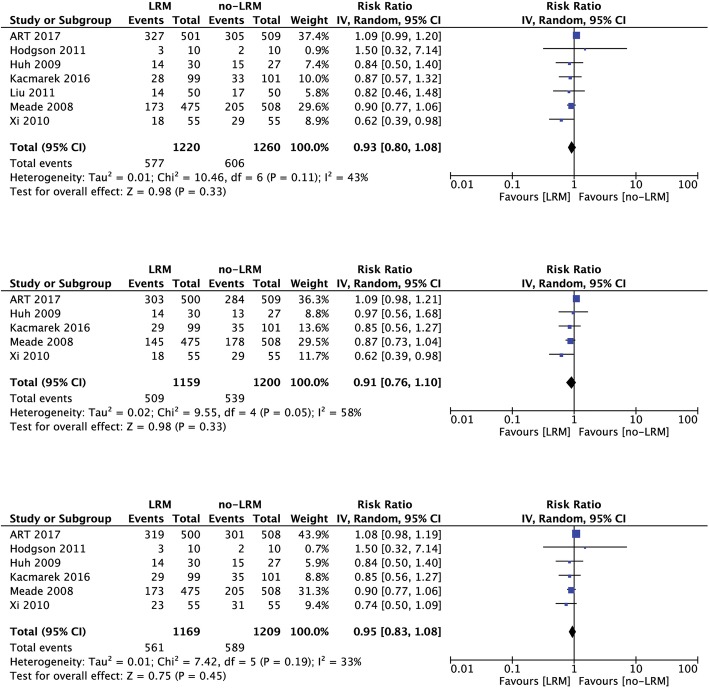
Forest plot showing odds ratio of **a** mortality at longest available follow-up at individual study level and pooled analysis level (upper); **b** ICU mortality at individual study level and pooled analysis level (middle); **c** in-hospital mortality at individual study level and pooled analysis level (lower)

Reported mortality at the longest available follow-up [RR (95% CI) 0.93 (0.80, 1.08); *p* = 0.33; *I*^2^ = 43%; *n* = 2480], ICU mortality [RR (95% CI) 0.91 (0.76, 1.10); *p* = 0.33; *I*^2^ = 58%; *n* = 2359] and in-hospital mortality [RR (95% CI) 0.95 (0.83, 1.08); *p* = 0.45, *I*^2^ = 33%; *n* = 2378] were similar between recruitment maneuver group and standard lung protective ventilation group. A forest plot for odds ratio of mortality at different time points at individual study level and pooled analysis level has been provided in Fig. 3. Visual inspection of funnel plot for publication bias revealed that included trials are near the apex of the arbitrary triangle; hence, possibilities of publication bias cannot be excluded here. Similar results were obtained when the trial by Xi et al. [16] was excluded as PEEP titration was not used along with recruitment maneuver in that study.

### Length of stay

**Fig. 4:**
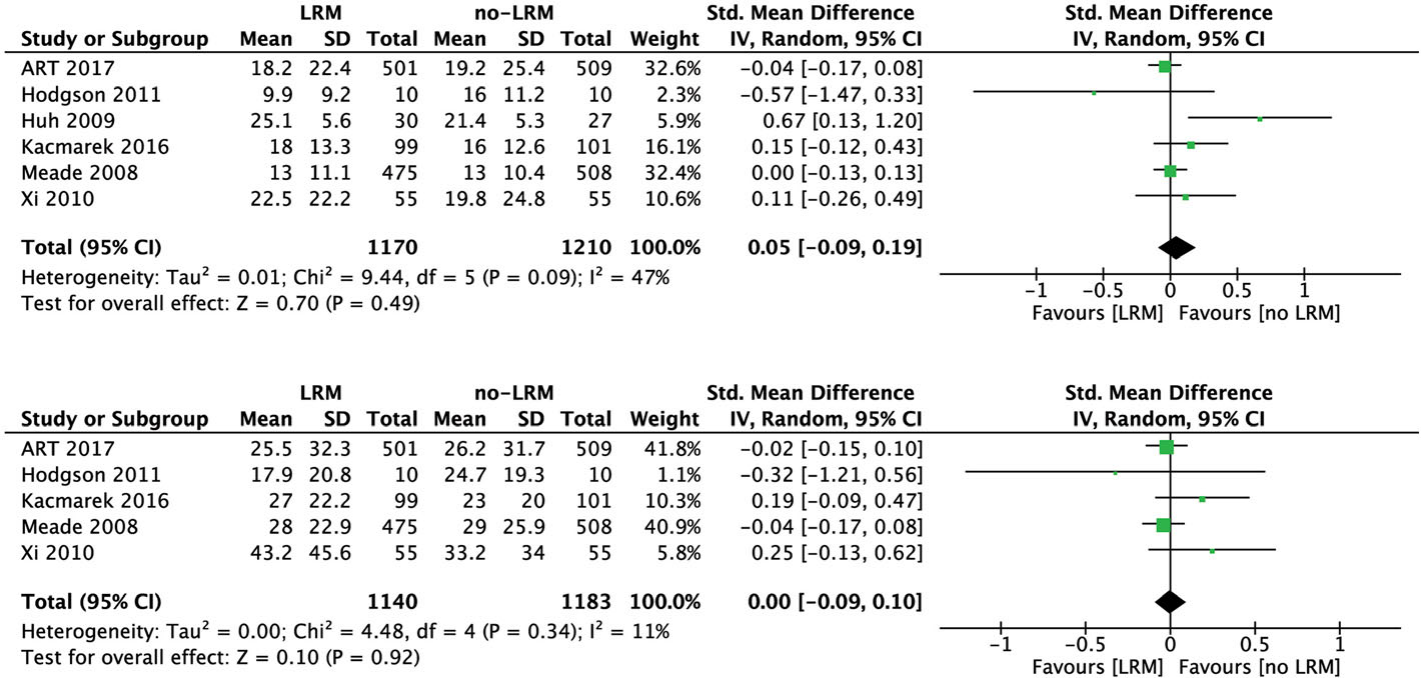
Forest plot showing standardized mean difference of **a** length of ICU stay (upper) and **b** length of hospital stay (lower) in two groups at individual study level and at the pooled analysis level

Duration of hospital stay [SMD (95% CI) 0.00 (− 0.09, 0.10); *p* = 0.92, *I*^2^ = 11%; *n* = 2323] and duration of ICU stays [SMD (95% CI) 0.05 (− 0.09, 0.19); *p* = 0.49, *I*^2^ = 47%; *n* = 2380] were similar between recruitment maneuver group and standard lung protective ventilation group. A forest plot for SMD in length of ICU stay and length of hospital stay at individual study level and pooled analysis level has been provided in Fig. 4. Similar results were obtained even after exclusion of the trial by Xi et al. [16].

### Complications

Only four trials reported incidence of barotrauma from recruitment maneuver, and it was found to be similar with standard lung protective ventilation group [RR (95% CI) 1.27 (0.68, 2.36); *p* = 0.45, *I*^2^ = 57%, *n* = 2350].

## Discussion

Principal findings of this meta-analysis and systematic review are that recruitment maneuver neither provides any mortality benefit nor reduces length of hospital and ICU stays in adult patients with ARDS. Findings of this meta-analysis contradicts the reported mortality benefits of recruitment maneuver by Goligher et al. [6] in a meta-analysis of randomized controlled trials that included 1423 patients from 6 trials. However, the authors did not include a recent large trial [11], and on the other hand, they included another trial, which did not use lung protective ventilation strategy in the control group [18]. In the light of present clinical knowledge, we believe that lung protective ventilation strategy is an integral part of ARDS management and studies those are not using it is at significant high risk of bias.

Another Cochrane database systematic review [22] reported a reduction in ICU mortality rate from a pooled analysis of data of 1370 patients from 5 trials. However, the authors did not report reduction in mortality in any other time points. Authors of that review of graded the quality of evidence as ‘low’ because of 4 of the included trials used various co-interventions along with recruitment maneuver. Though the co-interventions such as high PEEP or PEEP titration, used in various trials have the potential to interfere with the clinical outcome; from a physiological point of view, co-interventions to keep the recruited alveoli ‘open’ is an integral part of this approach. PEEP applied after recruitment maneuver expected to reduce sheer stress generated the collapsed and open alveoli interface from repeated recruitment and de-recruitment [23]. A higher PEEP with lung protective ventilation strategy may be beneficial in patients with ARDS [24].

Observational studies have found benefits of recruitment maneuver in ARDS patients in terms of oxygenation and lung compliance [25, 26]. An optimum PEEP and sigh maneuver also increases efficacy of recruitment maneuver in ARDS patients [27]. Toth et al. in 2007 suggested that improvement in oxygenation after recruitment maneuver and PEEP is due to primarily reduction in atelectasis rather than reduction in extra-vascular lung water [28]. However, success of recruitment maneuver may be dependent upon the amount of lung tissue available for recruitment and which is variable between patient and patient. In early ARDS, it may be possible to recruit lung and reverse hypoxemia in most of the patients [7]. Success of PEEP-induced recruitment may also depend upon the regional distribution and characteristics of the atelectasis and it may be greater in case of inflammatory atelectasis at the lower lobes [29].

In this meta-analysis, we have found that recruitment maneuver used along with or without PEEP titration does not provide any mortality benefit at any time points. Our results remain essentially similar even when the trial by Xi et al. [16] excluded, as they did not use any co-intervention along with recruitment maneuver. However, the Xi et al. reported a reduction in ICU mortality but not in hospital mortality with the standalone use of recruitment maneuver. These findings suggest that recruitment maneuver without PEEP titration might have some beneficial effect in ARDS patients.

### Limitations

Our meta-analysis has several limitations. We have found significant amount of statistical heterogeneity most of the all analyses which is probably due to a heterogeneity in patients’ selection and in the methods of recruitment maneuver application across the studies. As the number of the included trials were small in our meta-analysis, a meta-regression analysis was not possible. A visual inspection of the funnel plot also suggested that publication biases might also be present.

## Conclusion

Recruitment maneuver along with co-interventions such as PEEP titration does not provide any benefit in terms of mortality, length of ICU and hospital stay. Further studies are required to know the clinical benefits of recruitment maneuver without PEEP titration in ARDS patients.

## Additional file

Additional file 1: Appendix. (DOCX 63 kb)

## Abbreviations

95% CI: 95% confidence interval
ARDS: Acute respiratory distress syndrome
CENTRAL: The Cochrane Central Register of controlled trials
ICU: Intensive care unit
NNT: Number needed to treat
OR: Odds ratio
PaO_2_/FiO_2_: Ratio of arterial partial pressure of oxygen to fractional-inspired oxygen content
PEEP: Positive end-expiratory pressure
RCT: Randomized controlled trial
SD: Standard deviation
SMD: Standardized mean difference

## References

[1] Maitra S, Bhattacharjee S, Khanna P, Baidya DK (2015). High-frequency ventilation does not provide mortality benefit in comparison with conventional lung-protective ventilation in acute respiratory distress syndrome: a meta-analysis of the randomized controlled trials. Anesthesiology.

[2] McNicholas BA, Rooney GM, Laffey JG (2018). Lessons to learn from epidemiologic studies in ARDS. Curr Opin Crit Care..

[3] Bellani G, Laffey JG, Pham T, Fan E, Brochard L, Esteban A, Gattinoni L, van Haren F, Larsson A, McAuley DF, Ranieri M, Rubenfeld G, Thompson BT, Wrigge H, Slutsky AS (2016). Pesenti A; LUNG SAFE Investigators; ESICM Trials Group. Epidemiology, patterns of care, and mortality for patients with acute respiratory distress syndrome in intensive care units in 50 countries. JAMA.

[4] Albert RK (2012). The role of ventilation-induced surfactant dysfunction and atelectasis in causing acute respiratory distress syndrome. Am J Respir Crit Care Med.

[5] Pierrakos C, Karanikolas M, Scolletta S, Karamouzos V, Velissaris D (2012). Acute respiratory distress syndrome: pathophysiology and therapeutic options. J Clin Med Res.

[6] Goligher EC, Hodgson CL, Adhikari NKJ, Meade MO, Wunsch H, Uleryk E, Gajic O, Amato MPB, Ferguson ND, Rubenfeld GD, Fan E (2017). Lung recruitment maneuvers for adult patients with acute respiratory distress syndrome. A systematic review and meta-analysis. Ann Am Thorac Soc.

[7] Borges JB, Okamoto VN, Matos GF, Caramez MP, Arantes PR, Barros F, Souza CE, Victorino JA, Kacmarek RM, Barbas CS, Carvalho CR, Amato MB (2006). Reversibility of lung collapse and hypoxemia in early acute respiratory distress syndrome. Am J Respir Crit Care Med.

[8] Guo L, Wang W, Zhao N, Guo L, Chi C, Hou W, Wu A, Tong H, Wang Y, Wang C, Li E (2016). Mechanical ventilation strategies for intensive care unit patients without acute lung injury or acute respiratory distress syndrome: a systematic review and network meta-analysis. Crit Care.

[9] Liberati A, Altman DG, Tetzlaff J, Mulrow C, Gøtzsche PC, Ioannidis JP, Clarke M, Devereaux PJ, Kleijnen J, Moher D (2009). The PRISMA statement for reporting systematic reviews and meta-analyses of studies that evaluate health care interventions: explanation and elaboration. J Clin Epidemiol.

[10] Hozo SP, Djulbegovic B, Hozo I (2005). Estimating the mean and variance from the median, range, and the size of a sample. BMC Med Res Methodol.

[11] Cavalcanti AB, Suzumura ÉA, Laranjeira LN, Paisani DM, Damiani LP, Guimarães HP, Romano ER, Regenga MM, LNT T, Teixeira C, Pinheiro de Oliveira R, Machado FR, Diaz-Quijano FA, MSA F, Maia IS, Caser EB, Filho WO, Borges MC, Martins PA, Matsui M, Ospina-Tascón GA, Giancursi TS, Giraldo-Ramirez ND, SRR V, MDGPL A, Hasan MS, Szczeklik W, Rios F, MBP A, Berwanger O, Ribeiro de Carvalho CR, Writing Group for the Alveolar Recruitment for Acute Respiratory Distress Syndrome Trial (ART) Investigators (2017). Effect of lung recruitment and titrated positive end-expiratory pressure (PEEP) vs low PEEP on mortality in patients with acute respiratory distress syndrome: a randomized clinical trial. JAMA.

[12] Kacmarek RM, Villar J, Sulemanji D, Montiel R, Ferrando C, Blanco J, Koh Y, Soler JA, Martínez D, Hernández M, Tucci M, Borges JB, Lubillo S, Santos A, Araujo JB, Amato MB, Suárez-Sipmann F (2016). Open lung approach network. Open lung approach for the acute respiratory distress syndrome: a pilot, randomized controlled trial. Crit Care Med.

[13] Hodgson CL, Tuxen DV, Davies AR, Bailey MJ, Higgins AM, Holland AE, Keating JL, Pilcher DV, Westbrook AJ, Cooper DJ, Nichol AD (2011). A randomised controlled trial of an open lung strategy with staircase recruitment, titrated PEEP and targeted low airway pressures in patients with acute respiratory distress syndrome. Crit Care.

[14] Meade MO, Cook DJ, Guyatt GH, Slutsky AS, Arabi YM, Cooper DJ, Davies AR, Hand LE, Zhou Q, Thabane L, Austin P, Lapinsky S, Baxter A, Russell J, Skrobik Y, Ronco JJ, Stewart TE (2008). Lung Open Ventilation Study Investigators. Ventilation strategy using low tidal volumes, recruitment maneuvers, and high positive end-expiratory pressure for acute lung injury and acute respiratory distress syndrome: a randomized controlled trial. JAMA.

[15] Huh JW, Jung H, Choi HS, Hong SB, Lim CM, Koh Y (2009). Efficacy of positive end-expiratory pressure titration after the alveolar recruitment manoeuvre in patients with acute respiratory distress syndrome. Crit Care.

[16] Xi XM, Jiang L, Zhu B, RM group (2010). Clinical efficacy and safety of recruitment maneuver in patients with acute respiratory distress syndrome using low tidal volume ventilation: a multicenter randomized controlled clinical trial. Chin Med J (Engl).

[17] Liu W-L, Wang C-M, Chen W-L (2011). Effects of recruitment maneuvers in patients with early acute lung injury and acute respiratory distress syndrome. Respirology.

[18] Amato MB, Barbas CS, Medeiros DM, Magaldi RB, Schettino GP, Lorenzi-Filho G, Kairalla RA, Deheinzelin D, Munoz C, Oliveira R, Takagaki TY, Carvalho CR (1998). Effect of a protective-ventilation strategy on mortality in the acute respiratory distress syndrome. N Engl J Med.

[19] Oczenski W, Hörmann C, Keller C, Lorenzl N, Kepka A, Schwarz S, Fitzgerald RD (2004). Recruitment maneuvers after a positive end-expiratory pressure trial do not induce sustained effects in early adult respiratory distress syndrome. Anesthesiology.

[20] Wang Z, Zhu X, Li H, Wang T, Yao G (2009). A study on the effect of recruitment maneuver imposed on extravascular lung water in patients with acute respiratory distress syndrome. Chinese Critical Care Medicine.

[21] Yang G, Wang C, Ning R (2011). Effects of high positive end-expiratory pressure combined with recruitment maneuvers in patients with acute respiratory distress syndrome. Chinese Critical Care Med.

[22] Hodgson C, Goligher EC, Young ME, Keating JL, Holland AE, Romero L, Bradley SJ, Tuxen D (2016). Recruitment manoeuvres for adults with acute respiratory distress syndrome receiving mechanical ventilation. Cochrane Database Syst Rev.

[23] Slutsky AS (1999). Lung injury caused by mechanical ventilation. Chest.

[24] Briel M, Meade M, Mercat A, Brower RG, Talmor D, Walter SD, Slutsky AS, Pullenayegum E, Zhou Q, Cook D, Brochard L, Richard JC, Lamontagne F, Bhatnagar N, Stewart TE, Guyatt G (2010). Higher vs lower positive end-expiratory pressure in patients with acute lung injury and acute respiratory distress syndrome: systematic review and meta-analysis. JAMA.

[25] Póvoa P, Almeida E, Fernandes A, Mealha R, Moreira P, Sabino H (2004). Evaluation of a recruitment maneuver with positive inspiratory pressure and high PEEP in patients with severe ARDS. Acta Anaesthesiol Scand.

[26] Gernoth C, Wagner G, Pelosi P, Luecke T (2009). Respiratory and haemodynamic changes during decremental open lung positive end-expiratory pressure titration in patients with acute respiratory distress syndrome. Crit Care.

[27] Badet M, Bayle F, Richard JC, Guérin C (2009). Comparison of optimal positive end-expiratory pressure and recruitment maneuvers during lung-protective mechanical ventilation in patients with acute lung injury/acute respiratory distress syndrome. Respir Care.

[28] Toth I, Leiner T, Mikor A, Szakmany T, Bogar L, Molnar Z (2007). Hemodynamic and respiratory changes during lung recruitment and descending optimal positive end-expiratory pressure titration in patients with acute respiratory distress syndrome. Crit Care Med.

[29] Puybasset L, Gusman P, Muller JC, Cluzel P, Coriat P, Rouby JJ (2000). Regional distribution of gas and tissue in acute respiratory distress syndrome. III. Consequences for the effects of positive end-expiratory pressure. CT scan ARDS study group. Adult respiratory distress syndrome. Intensive Care Med.

