# Supplementary material for: Recruitment maneuver does not provide any mortality benefit over lung protective strategy ventilation in adult patients with acute respiratory distress syndrome: a meta-analysis and systematic review of the randomized controlled trials
Source: J Intensive Care. 2018 Jun 26;6:35. doi: 10.1186/s40560-018-0305-9 (PMC6019312; doi:10.1186/s40560-018-0305-9)
Supplement: Supplementary file 1 — Appendix. (DOCX 63 kb) [file 40560_2018_305_MOESM1_ESM.docx]

ARDS[All Fields]

"respiratory distress syndrome, adult"[MeSH Terms] OR ("respiratory"[All Fields] AND "distress"[All Fields] AND "syndrome"[All Fields] AND "adult"[All Fields]) OR "adult respiratory distress syndrome"[All Fields] OR ("acute"[All Fields] AND "respiratory"[All Fields] AND "distress"[All Fields] AND "syndrome"[All Fields]) OR "acute respiratory distress syndrome"[All Fields]

"acute lung injury"[MeSH Terms] OR ("acute"[All Fields] AND "lung"[All Fields] AND "injury"[All Fields]) OR "acute lung injury"[All Fields]

acute[All Fields] AND hypoxemic[All Fields] AND ("respiratory insufficiency"[MeSH Terms] OR ("respiratory"[All Fields] AND "insufficiency"[All Fields]) OR "respiratory insufficiency"[All Fields] OR ("respiratory"[All Fields] AND "failure"[All Fields]) OR "respiratory failure"[All Fields])

Recruitment [All Fields] AND maneuver[All Fields]

recruitment[All Fields] AND manoeuvre[All Fields]

("lung"[MeSH Terms] OR "lung"[All Fields]) AND recruitment[All Fields]
